# A Papillary Thyroid Cancer Metastasis Presented as a Ciliated Cyst in the Neck: An Unusual Presentation

**DOI:** 10.1002/ccr3.70935

**Published:** 2025-09-21

**Authors:** Ali Asilian, Mohammad Shoushtarizadeh, Sarah Seyedyousefi

**Affiliations:** ^1^ Skin Diseases and Leishmaniasis Research Center Isfahan University of Medical Sciences Isfahan Iran

**Keywords:** cyst, dermatology, dermatosurgery, pathology, thyroid

## Abstract

This case highlights the diagnostic challenges of neck masses that appear benign but are malignant upon histological examination. It underscores the need for comprehensive diagnostic procedures to rule out cancer. The atypical presentation of papillary thyroid carcinoma in this case underscores the importance of thorough evaluation of cervical masses to ensure accurate diagnosis and appropriate treatment.

## 1 Introduction

In adults, cervical masses are a significant clinical concern that often requires a thorough investigation to determine their origin. Although many cervical masses may be benign, like reactive lymphadenopathy or benign neoplasms, a significant number can be malignant, representing primary cancers or, more commonly, metastatic deposits from distant primary sites. The upper aerodigestive tract is a common source of such metastases, especially in individuals with a history of tobacco use or alcohol consumption. These risk factors greatly increase the chances of malignancy in middle‐aged and elderly patients with cervical masses. It is crucial to promptly and accurately identify the primary tumor to start effective treatment and enhance patient outcomes. Non‐invasive diagnostic methods are preferred to avoid the complications linked with open biopsy procedures. A comprehensive assessment typically involves imaging studies, fine‐needle aspiration cytology (FNAC), and molecular diagnostic tools [1, 2, 3]. The majority of lateral neck masses in individuals over the age of 40 are attributed to malignant tumors, with the prevalence of neoplastic cervical adenopathy rising as individuals grow older [4]. Some studies have demonstrated that 40% of these patients were diagnosed with metastatic squamous cell carcinoma originating from unknown primary sites. Lymphoma accounted for 39.5% of the cases, while the remaining patients had either benign disease (16.5%), sarcoma (2%), or chemodectomas (2%). Numerous researchers have conducted investigations to determine the source of metastatic squamous cell carcinoma in individuals with enlarged cervical lymph nodes [5, 6, 7]. The neck's clinically significant area is divided into two sections by the sternocleidomastoid muscle, namely the anterior and posterior triangles. While neck masses are less commonly found in the posterior triangle, malignancy tends to occur relatively more often in this particular region [8]. Anyhow, it is widely acknowledged in the literature that any enduring asymmetrical mass in the neck of an adult should be considered malignant unless there is conclusive evidence to prove otherwise [8].

Here we present a case of lateral neck mass without any characteristics of malignancy, which turned out to be malignant after pathologic investigations.

## 2 Case Presentation

### 2.1 Case History

A 48‐year‐old non‐smoking male patient was referred to the skin tumor clinic due to a lump on the right lateral side of his neck. The lump had been present for approximately 6 months, initially small but gradually growing in size. The patient reported that the lump was painless and had not changed in terms of symptoms over time. Upon physical examination, a cystic protrusion approximately 4 cm in diameter was observed on the right lateral side of the lower part of the neck. The mass was soft, non‐erythematous, non‐tender, and had a normal body temperature. It was mobile and not attached to the surrounding skin structure. There was no purulent or bloody discharge, and the skin covering the lesion appeared normal.

The patient had no significant medical history and was not taking any medication at the time of the onset of the symptoms. There was no family history of any type of malignancy or similar lesion.

### 2.2 Differential Diagnosis, Investigations, and Treatment

**FIGURE 1:**
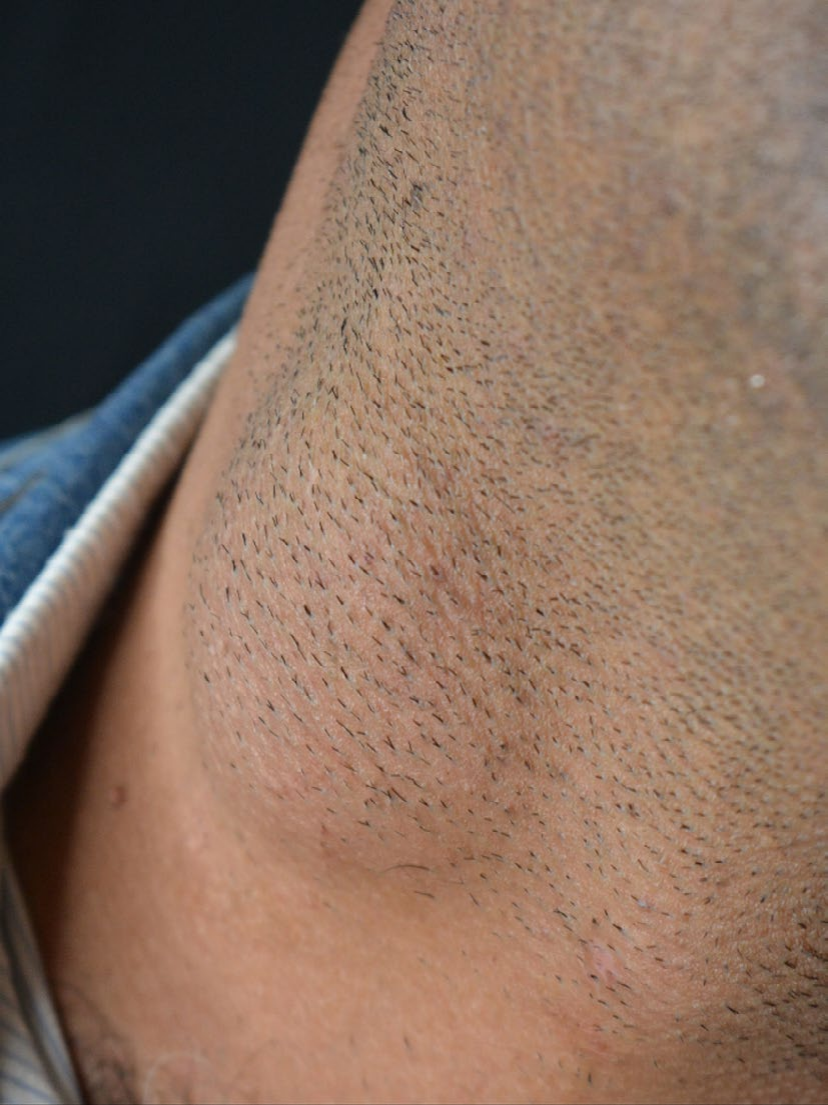
The mass was located on the right lateral side of the lower part of the neck.

The patient's symptoms and physical examination findings were consistent with a benign lesion, possibly a cyst or a lipoma. Further evaluation, including imaging studies and biopsy, seemed necessary to confirm the diagnosis and rule out any potential malignancy (Figure 1).

A sonographic examination of the mass was conducted and revealed a cystic lesion located anteriorly within the upper half of the sternocleidomastoid muscle. The lesion exhibited sharp borders and measured 46 mm in length, 49 mm in width, and 31 mm in height, with an approximate volume of 38 cm^3^. The cystic mass contained echogenic deposits and echogenic septa, including a complex internal structure. Notably, there was no evidence of a solid component or connection to the surrounding neck vessels. The imaging findings collectively suggested a diagnosis of bronchial cysts. The absence of a solid component and the cyst's location within the sternocleidomastoid muscle supported this diagnosis. To confirm the diagnosis, the patient was advised to take an excisional biopsy. After making sure that the cyst is not related to the vessels, the patient became a candidate for cyst excisional surgery.

**FIGURE 2:**
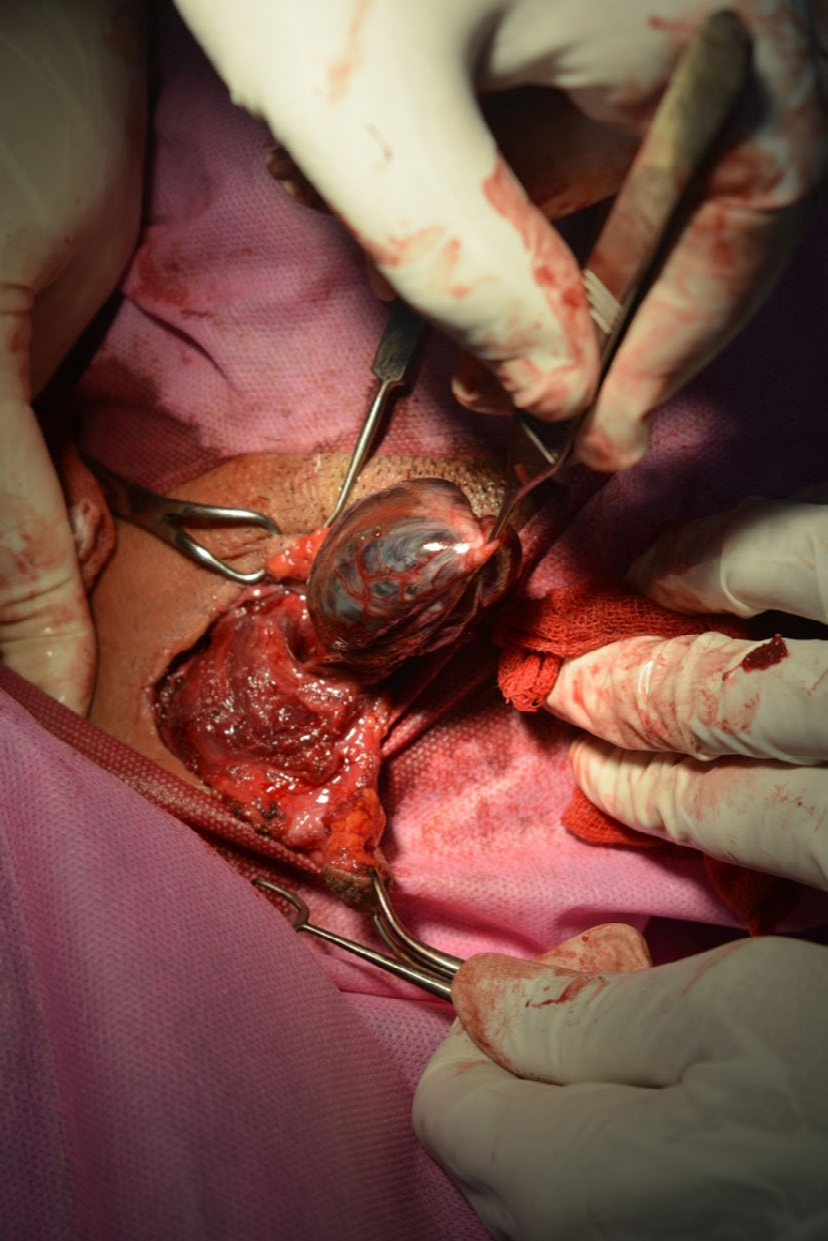
A cystic mass 3 cm in diameter and with brown color was excised successfully.

During the operation, a cystic mass 3 cm in diameter was removed. The wall thickness was estimated at about 0.1 cm, and careful examination of the cyst revealed papillated walls (Figure 2).

The mass, believed to be a ciliated cyst, underwent pathological examination for additional assessment.

**FIGURE 3:**
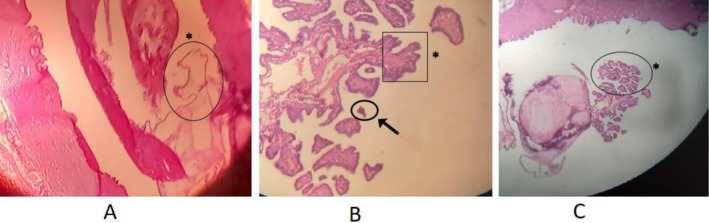
(A) Cervical lymph node metastasis of papillary thyroid carcinoma, cystic degeneration (H&E 4×). (B) Papilla having fibrovascular core (asterisk) and calcification (arrow) in the stroma. (H&E 4×). (C) Papilla lined by columnar cells with enlarged overlapped nuclei. (H&E 10×).

Upon microscopic examination, it was revealed that there was cystic degeneration present, and the papillae were covered by a layer of stratified columnar epithelium with ground glass nuclei and calcification. This finding was in line with the cystic degeneration of lymph node metastasis caused by papillary thyroid carcinoma. (Figure 3A,B,C).

A full thyroid evaluation was conducted and the patient had a TSH level of 38.1 in the workup. Finally, a papillary thyroid cancer was diagnosed by imaging and fine needle aspiration of the thyroid. Eventually, the patient was referred to endocrinological cancer experts and underwent lobar thyroidectomy.

After following up with the patient, there has been no evidence of metastasis or tumor recurrence.

## 3 Discussion

This article presented a case of a patient who exhibited a mass in the lateral region of the neck, which upon thorough examination and review of medical history displayed no indicators of malignancy. The absence of any previous malignancy in the patient during the initial evaluation is particularly noteworthy, and the characteristics of the mass post‐excision did not indicate malignancy either. However, after the excision of the mass and subsequent microscopic analysis, malignancy was confirmed. It is important to be suspicious of malignancy in any patient with a neck mass, especially in older patients.

Whenever a surgeon encounters a neck mass, numerous inquiries arise in their mind. These include determining whether it is congenital or acquired, inflammatory or non‐inflammatory, the tissue of origin, whether it is benign or malignant, and a primary or secondary lesion. If it is a secondary lesion, the surgeon must identify the source of the primary lesion or consider the possibility of an occult primary malignancy. Additionally, they must ascertain the necessary diagnostic tools and appropriate treatment for the condition [9]. There were multiple cases of benign irregular masses such as ciliated cysts in the neck area which were unusual, such as an epidermoid cyst in the parotid gland or masses related to the thyroglossal duct [10]. At the same time, sometimes masses in the neck are misdiagnosed as benign because they are only an unusual presentation of tumors such as schwannoma and are not expected in the neck region [11]. Accurate medical history and thorough physical examination are vital in gathering data, and the precise location of the mass and the progression over time can be essential in distinguishing neoplastic conditions from other potential diagnoses as well. Furthermore, the age of the patient can be a significant determining factor [12]. Neck ultrasound is typically conducted to offer high‐quality imaging of superficial structures, making it valuable for assessing the most detectable masses in the neck. Additionally, the evaluation should encompass the submandibular, parotid, and thyroid glands, as necessary. Recognizing a healthy thyroid gland is crucial in the preoperative assessment of certain congenital neck masses like thyroglossal duct cysts or ectopic thyroid [13]. It has been previously determined that regional spread from PTC typically occurs in the central area [14]. Sivanandan et al. [15] were the pioneers in outlining the lateral neck levels frequently affected by PTC. Kupferrman et al. discovered a notable proportion of patients with metastatic disease confined to a single level, and they showcased the required surgical measures to eliminate the disease in the lateral neck region [16].

In our case of neck mass, although none of the mass characteristics were in favor of malignant cancer, the final diagnosis of papillary thyroid cancer was reported. It's notable that not even the clinical course or the location of the mass was usual for a thyroid cancer metastasis. Therefore, we find it crucial to draw attention to the possibility of an unpleasant diagnosis even in the most unlikely cases to avoid missing the vital information for the patient's proper treatment.

## 4 Conclusion

In conclusion, this case underscores the critical importance of maintaining a high index of suspicion for malignancy in patients presenting with neck masses, even when initial clinical evaluations suggest a benign condition. The unexpected diagnosis of papillary thyroid carcinoma in a cystic neck mass, despite its benign appearance and atypical presentation, highlights the necessity for thorough diagnostic workups, including imaging and histopathological analyses. Clinicians must remain watchful and consider the possibility of malignancy in persistent neck masses. This case serves as a reminder that atypical presentations can occur and that comprehensive evaluation is essential to avoid missing potentially serious diagnoses.

## Author Contributions

**Ali Asilian:** conceptualization, data curation, formal analysis, funding acquisition, investigation, resources, software, supervision, validation, visualization, writing – original draft. **Mohammad Shoushtarizadeh:** conceptualization, formal analysis, funding acquisition, supervision, validation, visualization. **Sarah Seyedyousefi:** project administration, writing – review and editing.

## Disclosure

The authors have nothing to report.

## Ethics Statement

This study has obtained ethical approval from the Isfahan University of Medical Sciences.

## Consent

Written informed consent was obtained from the patient to publish this report in accordance with the journal's patient consent policy.

## Conflicts of Interest

The authors declare no conflicts of interest.

## Data Availability

All data used and analyzed during this study are available from the corresponding author upon reasonable request.

## References

[1] M. A. Pynnonen , M. B. Gillespie , B. Roman , et al., “Clinical Practice Guideline: Evaluation of the Neck Mass in Adults,” Otolaryngology and Head and Neck Surgery 157, no. 2_suppl (2017): S1–S30. 10.1177/0194599817722550 28891406

[2] J. Haynes , K. R. Arnold , C. Aguirre‐Oskins , and S. Chandra , “Evaluation of Neck Masses in Adults,” American Family Physician 91, no. 10 (2015): 698–706. 25978199

[3] A. S. Jones , J. A. Cook , D. E. Phillips , and N. R. Roland , “Squamous Carcinoma Presenting as an Enlarged Cervical Lymph Node,” Cancer 72, no. 5 (1993): 1756–1761. 8348505 10.1002/1097-0142(19930901)72:5<1756::aid-cncr2820720540>3.0.co;2-5

[4] J. L. Lefebvre , B. Coche‐Dequeant , J. T. Van , E. Buisset , and A. Adenis , “Cervical Lymph Nodes From an Unknown Primary Tumor in 190 Patients,” American Journal of Surgery 160, no. 4 (1990): 443–446. 2221252 10.1016/s0002-9610(05)80562-8

[5] W. M. Mendenhall , A. A. Mancuso , J. T. Parsons , S. P. Stringer , and N. J. Cassisi , “Diagnostic Evaluation of Squamous Cell Carcinoma Metastatic to Cervical Lymph Nodes From an Unknown Head and Neck Primary Site,” Head & Neck 20, no. 8 (1998): 739–744. 9790297 10.1002/(sici)1097-0347(199812)20:8<739::aid-hed13>3.0.co;2-0

[6] P. M. Stell , R. P. Morton , and S. D. Singh , “Cervical Lymph Node Metastases: The Significance of the Level of the Lymph Node,” Clinical Oncology 9, no. 2 (1983): 101–107. 6883836

[7] M. Barakat , L. M. Flood , V. H. Oswal , and R. W. Ruckley , “The Management of a Neck Mass: Presenting Feature of an Asymptomatic Head and Neck Primary Malignancy?,” Annals of the Royal College of Surgeons of England 69, no. 4 (1987): 181–184. 3631877 PMC2498463

[8] T. J. McDevitt and M. J. Acquarelli , “Cervical Lymphadenopathy Originating in the Scalp,” Archives of Otolaryngology 82, no. 4 (1965): 412–414. 5857216 10.1001/archotol.1965.00760010414016

[9] F. Pacini , M. Schlumberger , H. Dralle , R. Elisei , J. W. Smit , and W. Wiersinga , “European Consensus for the Management of Patients With Differentiated Thyroid Carcinoma of the Follicular Epithelium,” European Journal of Endocrinology 154, no. 6 (2006): 787–803. 16728537 10.1530/eje.1.02158

[10] M. El Omri , G. Grassi , M. Bellakhdhr , L. Mesbah , W. Kermani , and M. Abdelkefi , “A Rare Entity in Salivary Gland Pathology: A Case Report of an Epidermoid Cyst in the Parotid Gland,” International Journal of Surgery Case Reports 124 (2024): 110359. 39342790 10.1016/j.ijscr.2024.110359 PMC11470506

[11] U. Singh , S. Roy , K. Gaurav , A. Anand , S. Qayoom , and A. A. Sonkar , “Adrenal Schwannoma Presenting as an Incidentaloma in a Patient With Uterine Fibroids and Cholelithiasis: A Rare Case Report,” Indian Journal of Surgical Oncology 15, no. Suppl 3 (2024): 395–399. 39328720 10.1007/s13193-024-01969-z PMC11422325

[12] A. C. Limardo , L. Blanco , J. Menendez , and A. Ortega , “Evaluation of Primary Lateral Neck Mass in Adults: Cross Sectional Study,” Journal of Surgery and Medicine 4, no. 10 (2020): 891–897.

[13] D. Koischwitz and N. Gritzmann , “Ultrasound of the Neck,” Radiologic Clinics of North America 38, no. 5 (2000): 1029–1045. 11054967 10.1016/s0033-8389(05)70219-0

[14] S. Noguchi , A. Noguchi , and N. Murakami , “Papillary Carcinoma of the Thyroid. I. Developing Pattern of Metastasis,” Cancer 26, no. 5 (1970): 1053–1060. 5476786 10.1002/1097-0142(197011)26:5<1053::aid-cncr2820260513>3.0.co;2-x

[15] R. Sivanandan and K. C. Soo , “Pattern of Cervical Lymph Node Metastases From Papillary Carcinoma of the Thyroid,” British Journal of Surgery 88, no. 9 (2001): 1241–1244. 11531874 10.1046/j.0007-1323.2001.01843.x

[16] M. E. Kupferman , M. Patterson , S. J. Mandel , V. LiVolsi , and R. S. Weber , “Patterns of Lateral Neck Metastasis in Papillary Thyroid Carcinoma,” Archives of Otolaryngology – Head & Neck Surgery 130, no. 7 (2004): 857–860. 15262763 10.1001/archotol.130.7.857

